# A Reduced-Acidification Phenotype Simplifies Strain Engineering in *Komagataeibacter* and Enables One-Step Production of Melanated Bacterial Cellulose

**DOI:** 10.3390/polym18172099

**Published:** 2026-08-29

**Authors:** Eric Handy, Andrea Shepard, Cecelia Kinane, Tanner Hamann, Megan Hannegan, Julie Gleason

**Affiliations:** Johns Hopkins Applied Physics Laboratory, Laurel, MD 20723, USA

**Keywords:** *Komagataeibacter sucrofermentans*, bacterial cellulose, phenotypic screening, glucose dehydrogenase, acidification, strain engineering, tyrosinase, melanin

## Abstract

*Komagataeibacter* species are among the highest-yielding bacterial cellulose producers and offer a promising platform for the genetic engineering of functionalized bacterial cellulose. However, routine strain engineering remains limited by inefficient screening of genomic integrants and acidic culture conditions that inhibit acid-sensitive cellulose modifications. Here, we exploited the reduced-acidification phenotype of a *Komagataeibacter sucrofermentans* glucose dehydrogenase deletion mutant (Δ*gdh*) to overcome both limitations. We developed a simple phenotypic screen based on reduced acidification to identify candidate colonies for subsequent molecular confirmation. We further exploited this phenotype by constructing a Δ*gdh*::*tyr1* strain that, after optimizing culture conditions, produced melanated bacterial cellulose in a single step, without the manual pH neutralization required by previous methods. Together, these results establish reduced acidification as a practical engineering phenotype that simplifies strain engineering and enables acid-sensitive modification of bacterial cellulose, thereby expanding the range of bacterial cellulose modifications achievable in *Komagataeibacter*.

## 1. Introduction

Bacterial cellulose has emerged as an attractive biomaterial for applications including textiles, composite materials, wound dressings, personal care products, and food additives due to its high crystallinity, flexibility, porosity, purity, and ease of production compared with plant-derived cellulose [1]. Considerable effort has focused on functionalizing bacterial cellulose to impart new properties, including antimicrobial activity [2], magnetic properties [3], bioelectric potential [4], and bioethanol purification [5]; however, these modifications typically require extensive downstream chemical processing. Genetic engineering of cellulose-producing microorganisms offers an alternative approach by enabling functionalization during cellulose biosynthesis. This one-step strategy could reduce the need for post-production chemical modification and simplify the production of functionalized bacterial cellulose.

Members of the genus *Komagataeibacter* are among the highest-yielding bacterial cellulose producers and have become important hosts for engineering functionalized bacterial cellulose [6]. Recent advances have produced strains with improved cellulose production [7], altered fiber morphology [8], and in vivo incorporation of chitin monomers [9]. At the same time, extensive genetic toolkits have expanded the available plasmids, promoters, reporters, and secretion systems for this genus [10,11].

Despite these advances, significant challenges remain in developing engineered bacterial cellulose materials. Conventional functionalization strategies often require extensive post-production processing, while practical limitations continue to reduce the efficiency of genetic engineering in *Komagataeibacter* and restrict implementation of desirable functional phenotypes. One of these is the difficulty of rapidly identifying correctly engineered transformants following genomic modification, possibly due to excessive cellulose, which inhibits DNA extraction [10] (Supplementary Information). Previous studies have demonstrated that deletion of the pyrroloquinoline quinone (PQQ)-dependent glucose dehydrogenase (*gdh*) reduces medium acidification and increases bacterial cellulose production in multiple *Komagataeibacter* species when grown on glucose [12,13]. The acidic extracellular environment characteristic of acetic acid bacteria also limits functionalization strategies that depend on acid-sensitive enzymes. One example is the production of melanated bacterial cellulose through expression of the *Bacillus megaterium* tyrosinase, encoded by *tyr1*, which has previously required manual pH neutralization following pellicle formation [14] because the enzyme is inhibited under acidic conditions [15]. These observations suggest that the reduced-acidification phenotype resulting from *gdh* deletion could provide a practical solution to both strain engineering and acid-sensitive functionalization.

In this study, we investigated whether the reduced-acidification phenotype resulting from *gdh* deletion could be exploited to address two practical challenges in engineering *Komagataeibacter*. We developed a rapid, inexpensive phenotype-based screening strategy for identifying engineered transformants and demonstrated that reduced acidification enabled one-step production of melanated bacterial cellulose without manual pH neutralization. Together, these findings establish reduced acidification as a practical engineering strategy that simplifies strain engineering while expanding opportunities for acid-sensitive bacterial cellulose functionalization.

## 2. Materials and Methods

### 2.1. Microbial Growth and Cellulose Production

*Komagataeibacter sucrofermentans* ATCC 700178 was obtained from the American Type Culture Collection (Manassas, VA, USA). Routine growth was performed in Hestrin-Schramm (HS) medium containing 5 g/L Phytone peptone (Becton Dickinson, Franklin Lakes, NJ, USA), 5 g/L yeast extract (Becton Dickinson, Franklin Lakes, NJ, USA), 2.7 g/L sodium phosphate dibasic heptahydrate (Thermo Fisher Scientific, Waltham, MA, USA), 1.5 g/L sodium citrate tribasic dihydrate (Sigma-Aldrich, Burlington, MA, USA), 0.6 g/L anhydrous magnesium sulfate (Thermo Fisher Scientific, Waltham, MA, USA), and either 20 g/L D-glucose (HSG; Sigma-Aldrich, Burlington, MA, USA) or 25 g/L D-mannitol (HSM; PhytoTech Labs, Lenexa, KS, USA). Where indicated, media were supplemented with 0.5 g/L L-tyrosine (HSGT or HSMT; Sunrise Science Products, Knoxville, TN, USA). Liquid media were supplemented with 5 U/mL cellulase from *Trichoderma reesei* (designated “-Cel”; Sigma-Aldrich, Burlington, MA, USA) and sterilized by filtration through a 0.22 μm syringe filter. Medium pH was adjusted to 5.7 with citric acid. Solid media contained 1.5% (*w*/*v*) agar, and all media were sterilized by autoclaving at 121 °C for 15 min. Routine starter cultures were grown overnight at 30 °C in 5 mL medium in 50 mL conical tubes.

### 2.2. Pellicle Production and Processing

Unless otherwise indicated, overnight starter cultures were washed twice with the appropriate cellulase-free medium, resuspended in the same medium, and inoculated into pellicle production media. Pellicles were incubated statically at 30 °C in a humidity-controlled chamber with 65–75% relative humidity. When required, pellicles were treated by incubation in two successive 1-h washes with 500 mL deionized water followed by a 2-h incubation in 0.1 M NaOH at 85 °C with shaking at 35 rpm. Following NaOH treatment, pellicles were washed twice with deionized water until the rinsate reached neutral pH, dried at 80 °C to constant weight, and weighed.

### 2.3. Genetic Engineering

DNA parts and primers used in this study are included in Table S1, and gBlock sequences are included in Table S2. pPETS_003 was constructed in pUC19, and pPETS_051 and pPETS_014 were constructed in pBBR1. NEB 5-alpha competent *E. coli* cells (catalog no. C2987H; New England Biolabs, Ipswich, MA, USA) were used for plasmid construction. A gBlock containing the *BBa_J23104* promoter, the *BBa_B0035* ribosomal binding site (RBS), the *tyr1* coding sequence from *Bacillus megaterium*, and the *rrnB1* terminator was ordered from Integrated DNA Technologies (Coralville, IA, USA). This sequence is the same as that used for *tyr1* expression in *K. rhaeticus* by Walker et al. [14]. All genetic components were amplified using the Platinum SuperFi II PCR Master Mix (catalog no. 12368050; Thermo Fisher Scientific; Waltham, MA, USA) and assembled using the NEBuilder HiFi DNA Assembly Master Mix (catalog no. E2621L; New England Biolabs; Ipswich, MA, USA). Plasmid sequences were verified by Oxford Nanopore Sequencing (Plasmidsaurus; South San Francisco, CA, USA).

**Figure 1:**
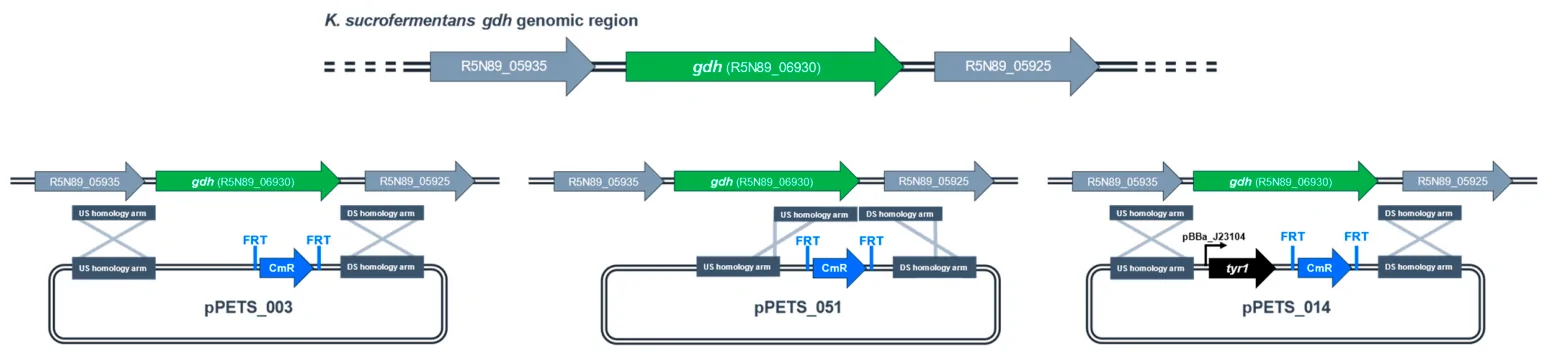
Construction of the Δ*gdh*, *gdh*+ (complemented Δ*gdh*), and Δ*gdh*::*tyr1* strains. The *K. sucrofermentans gdh* genomic region is shown at the top. Plasmids used for homologous recombination to generate the Δ*gdh* (pPETS_003), *gdh*+ (complement the Δ*gdh*) (pPETS_051), and construct the Δ*gdh*::*tyr1* strain (pPETS_014) are shown below. US, upstream homology; DS, downstream homology; CmR, chloramphenicol resistance cassette; FRT, FLP recombinase recognition target.

*K. sucrofermentans* electrocompetent cells were prepared as previously described [10] (Supplementary Information). Cells (100 µL) were transformed by electroporation (2485V, 50 µF, 100 Ω, 1 mm electrocuvette) with 1 μg PCR product composed of inserts of interest flanked by approximately 1 kb homology arms (Figure 1; Table S3). Transformants were recovered in 1 mL HSM-Cel overnight at 30 °C with 225 rpm shaking, and selected on HSG agar plates containing 350 μg mL^−1^ chloramphenicol (HSG-Cm) unless otherwise noted. Strains were screened for integration into the *gdh* locus as described and were verified by whole-genome sequencing (Plasmidsaurus; South San Francisco, CA, USA).

### 2.4. Colony PCR Evaluation

Five independent cultures each of wild-type (WT) *K. sucrofermentans* were grown in HSM-Cel for two days, and *E. coli* DH5α was grown in LB for one day. Cultures were adjusted to an OD_600_ of 0.1 and serially diluted to OD_600_ values of 0.01 and 0.001 in phosphate-buffered saline (PBS; catalog no. 10010023; Gibco, Waltham, MA, USA) or Phire Dilution Buffer (from the Thermo Scientific Phire Plant Direct PCR Master Mix, catalog no. F160L; Thermo Fisher Scientific, Waltham, MA, USA). One microliter of each suspension was used as a template for PCR using Hot Start Taq DNA Polymerase (catalog no. M0495L; New England Biolabs, Ipswich, MA, USA) or Phire Plant Direct PCR Master Mix (catalog no. F160L; Thermo Fisher Scientific, Waltham, MA, USA) according to the manufacturers’ instructions. Primers PETS_ETH_107/PETS_ETH_109 amplified a 940 bp region of the *E. coli groL* gene, whereas primers O_PETS_11/O_PETS_17 amplified a 1080 bp region upstream of the *K. sucrofermentans gdh* (Table S3). PCR products were separated on 1% agarose gels containing ethidium bromide.

As a positive control, *K. sucrofermentans* colony biomass was dispersed in PBS containing 10 U/mL cellulase for 2 h at room temperature, pelleted, resuspended in Phire Dilution Buffer, and boiled for 20 min. Following centrifugation, 1 μL of the supernatant was used as a template for PCR.

### 2.5. Phenotype-Based Transformant Screening

A pH-responsive indicator medium was prepared by supplementing HSG or HSM with 40 mg/L bromocresol green sodium salt (catalog no. A17503.06; Thermo Fisher Scientific, Waltham, MA, USA) and 80 mg/L thymol blue sodium salt (catalog no. 861367-5G; Sigma-Aldrich, Burlington, MA, USA) before autoclaving, yielding HSG-pH and HSM-pH, respectively. WT, Δ*gdh*, and *gdh*+ starter cultures were prepared as described in Section 2.1, adjusted to an OD_600_ of 1, and inoculated into duplicate 0.5 mL cultures of HSG-Cel, HSG-pH-Cel, HSM-Cel, or HSM-pH-Cel at an initial OD_600_ of 0.01 in 48-well plates. Plates were sealed with Breathe-Easier membrane (catalog no. 9126-2000; USA Scientific, Ocala, FL, USA) and incubated at 30 °C with shaking at 250 rpm for 7 days. Colorimetric pH standards were prepared by adjusting sterile HSG-pH and HSM-pH to pH values of 7.0, 6.0, 5.0, 4.0, 3.5, and 3.0 using hydrochloric acid or sodium hydroxide.

To validate phenotype-based transformant screening, equal optical densities of Δ*gdh* and *gdh*+ cultures were combined to generate a 1:1 mock community. Serial dilutions were plated on HSG-Cm-pH agar and incubated for 7 days. Twenty randomly selected colonies, together with known Δ*gdh* and *gdh*+ controls, were transferred to fresh HSG-Cm-pH agar and HSG-Cm-pH-Cel liquid medium, and colony identities were assigned based on medium color. PCR products were generated using primers O_PETS_11 and PETS_ETH_4 (Table S3), and selected amplicons were sequenced by Plasmidsaurus (South San Francisco, CA, USA). Sequence alignments were performed using SnapGene software Version 8.1 (GSL Biotech, San Diego, CA, USA) [16]. The screening workflow was subsequently applied during construction of the Δ*gdh::tyr1* strain by screening transformants on HSG-Cm-pH medium prior to whole-genome sequencing.

### 2.6. Establishing Conditions for One-Step Production of Melanated Bacterial Cellulose

Initial one-step melanation conditions were evaluated using previously reported culture conditions [14]. Δ*gdh* and Δ*gdh::tyr1* starter cultures were prepared as described above, adjusted to an OD_600_ of 1, and inoculated at an initial OD_600_ of 0.05 into duplicate 1 mL cultures of HSGT or HSMT supplemented with 10 μM CuSO_4_·5H_2_O (Thermo Fisher Scientific; Waltham, MA, USA) in 24-well plates. Cultures were incubated for 7 days as described in Section 2.2.

Melanation conditions were subsequently optimized by varying carbon source, CuSO_4_ concentration, and inoculation density. Δ*gdh* and Δ*gdh*::*tyr1* starter cultures were inoculated at initial OD_600_ values of 0.01, 0.05, or 0.1 into 0.5 mL HSGT or HSMT supplemented with 0, 10, 100, 250, 500, 750, or 1000 μM CuSO_4_·5H_2_O in 48-well plates. Cultures were incubated for 7 days as described in Section 2.2.

To evaluate the effect of copper pre-exposure, starter cultures were adjusted to an OD_600_ of 0.05, inoculated into 1 mL of HSMT-Cel containing 0, 7.5, 75, or 750 μM CuSO_4_·5H_2_O, and incubated for 2 days. Cells equivalent to 0.1 OD units were then washed twice with HSMT and inoculated into 1 mL of HSMT supplemented with 750 μM CuSO_4_·5H_2_O for pellicle production.

### 2.7. Characterization of Bacterial Cellulose

Pellicles produced by the WT, Δ*gdh*, and Δ*gdh*::*tyr1* strains under the optimized one-step melanation conditions described in Section 2.6 were processed as described in Section 2.2 and characterized by dry weight determination, Fourier transform infrared (FTIR) spectroscopy, and X-ray diffraction (XRD). FTIR was conducted using a Nicolet Summit FTIR (Thermo Fisher Scientific, Waltham, MA, USA) with a diamond attenuated total reflectance accessory. Each sample was measured in three places using 32 scans at 4 cm^−1^ resolution in the range of 4000 to 400 cm^−1^.

XRD patterns were measured with an Empyrean X-ray diffractometer (Malvern Panalytical, Malvern, United Kingdom) using Copper Kα_1_ radiation (1.540598 Å; 45 kV and 40 mA). XRD patterns were measured from 2θ = 5–50°, with scan steps of 0.017° at 0.5°/min. The Divergence Slit was 0.435°, the anti-scatter slit was 5.5 mm, and the Soller slits were set to 0.04 rad. The monochromator and collimator were not used. Dried cellulose films were mounted onto glass cover slides lightly coated in Vaseline to anchor the films, and the slides were mounted onto standard XRD sample holders. The X-ray background was subtracted from the measured XRD patterns before cellulose crystallinity analysis. Cellulose crystallinity was calculated using the Segal peak height method [17]:

(1) $$C_{r}I=\frac{\left(I_{200}-I_{am}\right)}{I_{200}}\times 100\%$$

where I_200_ and I_am_ are the background-subtracted peak intensities at 2θ = 22.2 and 18.6°, corresponding to the crystal plane (200) and amorphous region, respectively.

### 2.8. Statistical Analysis

All quantitative experiments were performed using three biological replicates unless otherwise noted. Values are reported as mean ± standard deviation (SD). Statistical significance was determined using two-way ANOVA followed by Tukey’s multiple-comparison test, with *p* < 0.05 considered statistically significant.

## 3. Results

### 3.1. Effect of Gdh Deletion on Cellulose Production

Preliminary evaluation of several carbon source formulations, including glucose, fructose, glycerol, sucrose, sorbitol, coconut water, sugar cane juice, and mannitol identified mannitol as the preferred carbon source for subsequent studies because it enabled robust bacterial cellulose production consistently (Table 1). Available wild-type growth curves and representative pellicle images for glucose and mannitol are provided in Figure S1. To establish the Δ*gdh* strain as the chassis for subsequent engineering, we next evaluated the effect of *gdh* deletion on bacterial cellulose production using glucose and mannitol as carbon sources (Table 1). Cellulose production was significantly greater on mannitol than on glucose regardless of strain. Although mean cellulose yields differed among strains grown on glucose, no significant differences were observed among strains grown on either carbon source. Δ*gdh* cultures produced robust pellicles on glucose; by contrast, pellicles produced by the WT and *gdh*+ strains were thin and fragile. Deletion of *gdh* did not affect cellulose production when mannitol was used as the carbon source. Together, these results establish the Δ*gdh* strain as a suitable engineering chassis by demonstrating that the reduced-acidification phenotype does not compromise bacterial cellulose production.

### 3.2. Standard Taq-Mediated Colony PCR Is Ineffective for K. sucrofermentans

**Figure 2:**
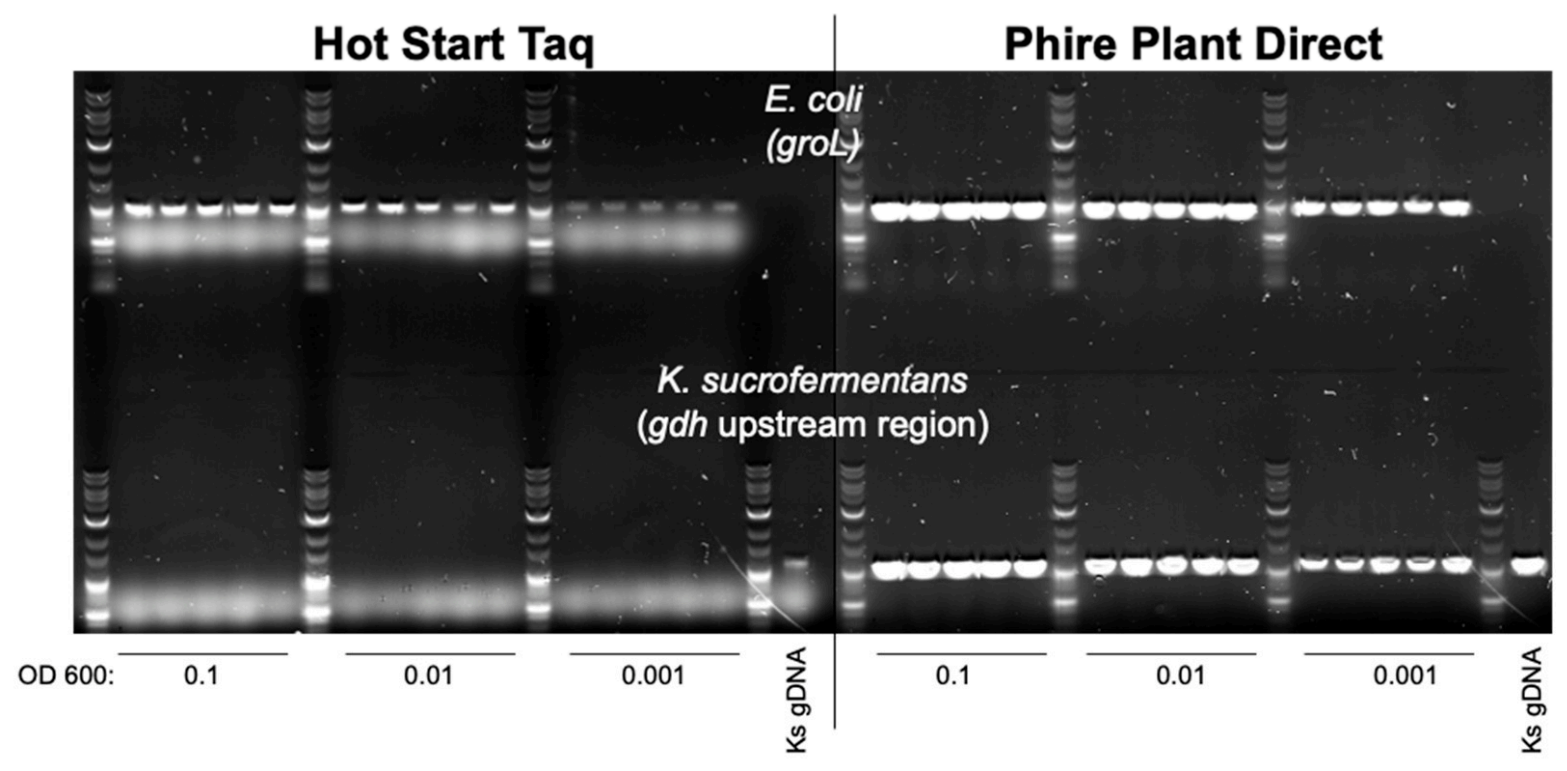
Performance of standard Taq-mediated colony PCR and Plant Phire Direct PCR using *E. coli* and *K. sucrofermentans* lysates. Lysates were prepared from cultures adjusted to an OD_600_ of 0.1, 0.01, and 0.001. Five independent PCR reactions were performed at each cell density using primers targeting the single-copy *groL* locus in *E. coli* and the *gdh* upstream region in *K. sucrofermentans*. PCR amplification was performed using NEB Hot Start Taq (**left**) or Thermo Scientific Plant Phire Direct PCR (**right**). Purified *K. sucrofermentans* genomic DNA (gDNA) served as a positive control.

Because efficient strain engineering depends on reliable identification of transformants, we evaluated the performance of the NEB Hot Start Taq and Thermo Scientific Plant Phire Direct PCR kits across a range of cell densities using *K. sucrofermentans* (Figure 2). Hot Start Taq failed to reproducibly amplify DNA from *K. sucrofermentans* regardless of cell density, whereas amplification was reproducible for *E. coli* and purified *K. sucrofermentans* genomic DNA. By comparison, the Plant Phire Direct PCR kit reproducibly amplified DNA from both organisms across all cell densities tested, but at approximately three-fold greater reagent cost per reaction. Together, these results demonstrate that standard Taq-mediated colony PCR is ineffective for *K. sucrofermentans*, motivating the development of an alternative phenotype-based screening strategy.

### 3.3. Development of a Phenotypic Screening Strategy Based on Reduced Acidification

**Figure 3:**
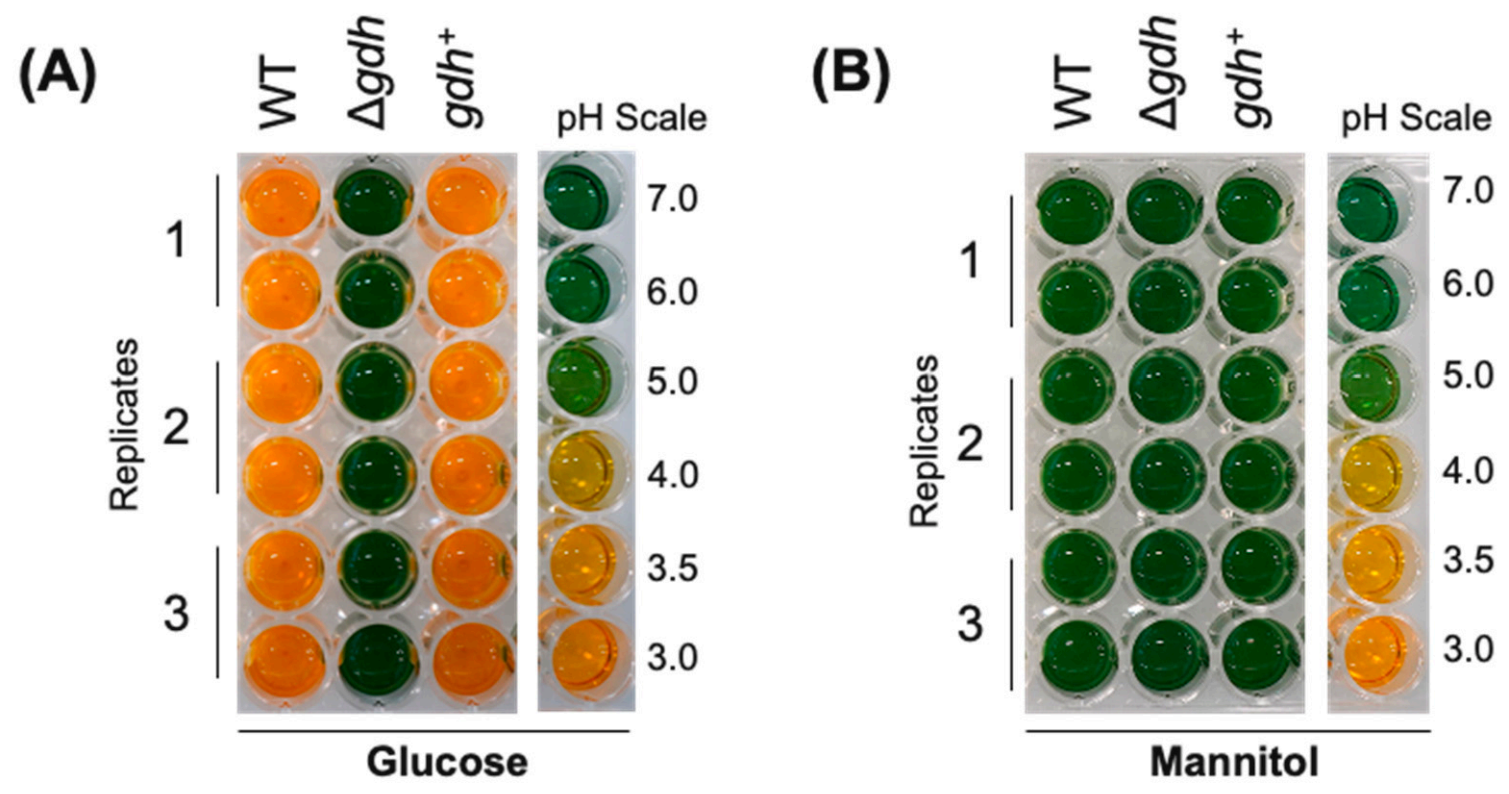
Colorimetric assay for distinguishing Δ*gdh* and *gdh*+ strains based on medium acidification. Wild-type (WT), Δ*gdh*, and *gdh*+ strains were inoculated into HS medium supplemented with bromocresol green and thymol blue and contained either (**A**) glucose or (**B**) mannitol as the sole carbon source. Three independent biological replicates are shown. A pH color scale is included for reference.

Because standard Taq-mediated proved unreliable for *K. sucrofermentans*, we investigated whether differential medium acidification could be used as a rapid, inexpensive method to screen transformants. Accordingly, restoration of *gdh* was expected to restore the acidification phenotype, providing a visual marker for transformant identification. To visualize these differences, HS medium supplemented with either glucose or mannitol was also supplemented with bromocresol green and thymol blue. When grown on glucose, the WT and *gdh*+ strains reproducibly acidified the medium from an initial pH of 5.7 to approximately pH 3.0—3.5, whereas the Δ*gdh* strain had little effect on medium pH (Figure 3A). By contract, no obvious differences in acidification were observed among strains grown on mannitol (Figure 3B), confirming that differential medium acidification is specific to *gdh*-dependent glucose metabolism and provides a suitable phenotype for transformant screening.

**Figure 4:**
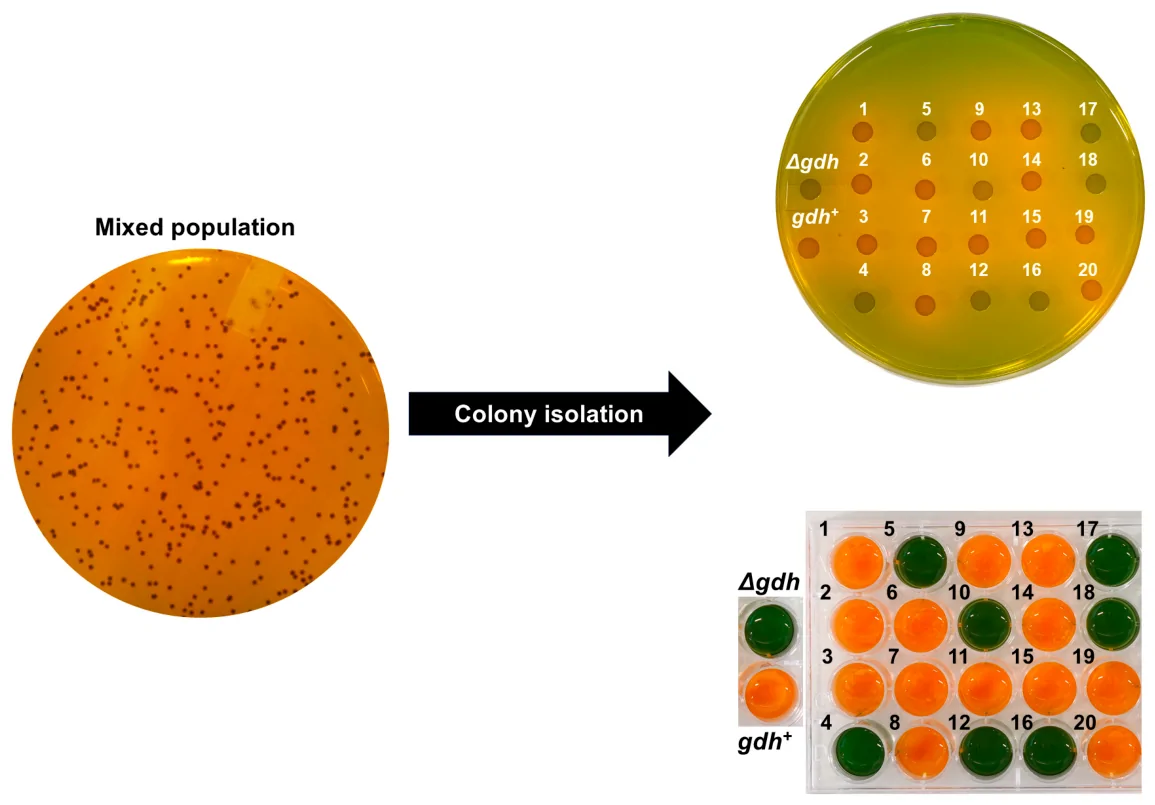
Phenotypic screening of *K. sucrofermentans* transformants based on differential medium acidification. A mixed population containing equal proportions of Δ*gdh* and *gdh*+ strains was plated on selective indicator agar. Following colony isolation, colonies were transferred to fresh indicator agar or liquid indicator medium (upper and lower right, respectively), where the acidification phenotype distinguished Δ*gdh* (orange) and *gdh*+ (green) isolates. Numbered isolates correspond to the molecular validation presented in Figure S2.

To determine whether this phenotype could be used to identify transformants, a mixed population containing equal proportions of Δ*gdh* and *gdh*^+^ strains was plated on selective indicator agar consisting of the HSG medium containing chloramphenicol, bromocresol green, and thymol blue. Colony identities could not be reliably distinguished directly on the mixed plate; however, following isolation onto fresh indicator agar and into the liquid indicator medium, the Δ*gdh* and *gdh*^+^ strains consistently produced distinct orange and green phenotypes, respectively (Figure 4), allowing candidate transformants to be readily distinguished following isolation.

The phenotype-based assignments were validated by colony PCR and amplicon sequencing (Table 2; Figure S2). Standard Hot Start Taq colony PCR yielded reproducible amplification for only 6 out of 20 isolates. By contrast, the Phire Plant Direct Kit increased the amplification success rate to 15 out of 20 isolates. For all isolates that yielded PCR products, amplicon sequencing confirmed that the molecular identification agreed with the phenotype-based assignments. Together, these results demonstrate that differential medium acidification provides a rapid, inexpensive, and reliable first-pass screen for identifying candidate *K. sucrofermentans* transformants for subsequent molecular confirmation.

### 3.4. One-Step Production of Melanated Bacterial Cellulose

**Figure 5:**
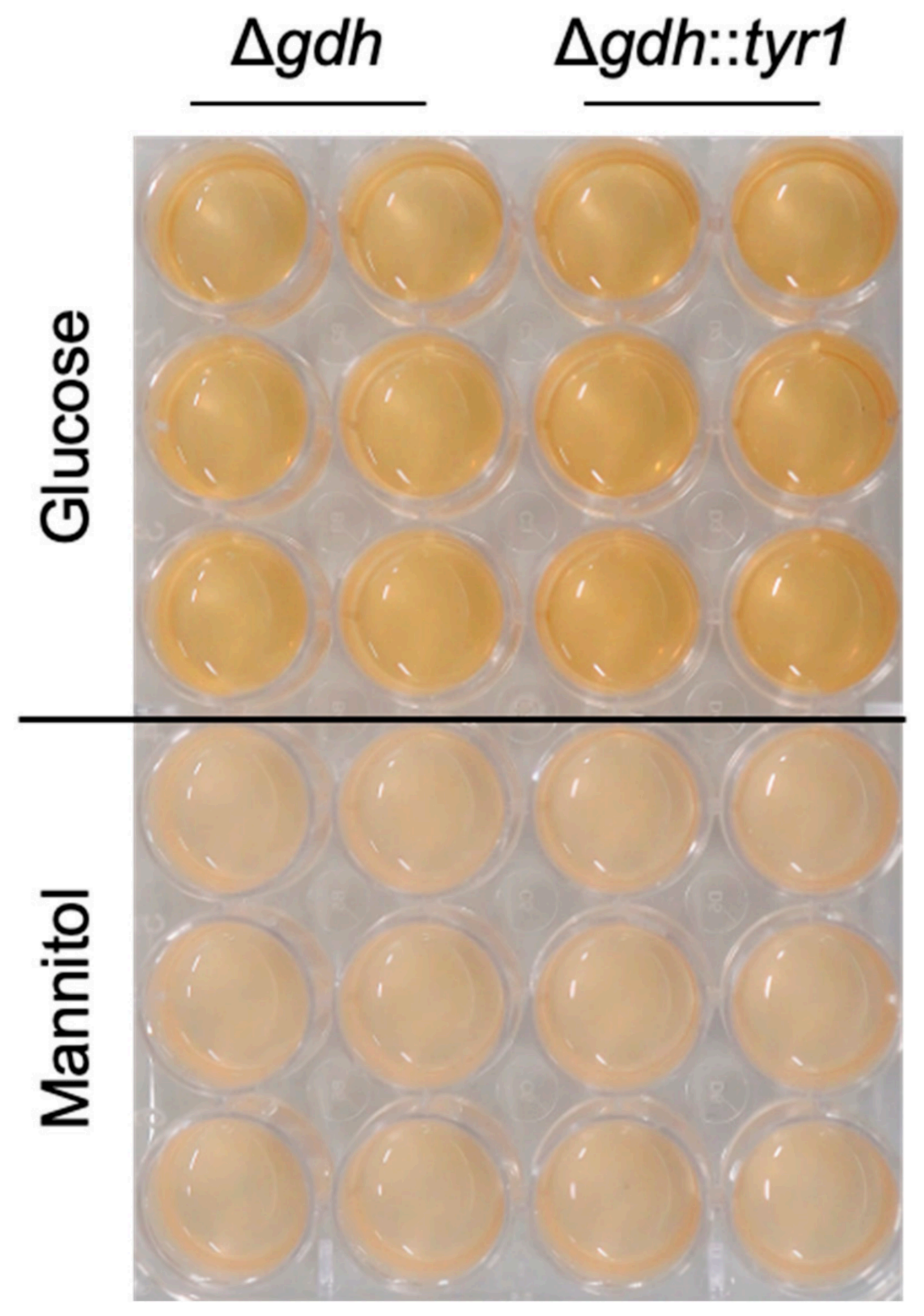
Initial evaluation of one-step melanated bacterial cellulose. Triplicate cultures of the Δ*gdh* and Δ*gdh*::*tyr1* strains were grown in HS medium containing either glucose or mannitol and supplemented with L-tyrosine (0.5 g/L) and CuSO_4_ (10 µM) under previously reported melanation conditions. No visually detectable melanin was observed in the Δ*gdh*::*tyr1* strain after one week of growth.

Having established the Δ*gdh* strain as an engineering chassis, we next investigated whether the reduced-acidification phenotype could enable acid-sensitive modifications of bacterial cellulose during pellicle formation. As a proof of concept, we chose melanin production because tyrosinase activity is inhibited under acidic conditions. To test this hypothesis, a Δ*gdh*::*tyr1* strain was constructed, identified using the phenotype-based screening described above (Figure S3), and confirmed by whole-genome sequencing. We first evaluated melanated pellicle production under previously reported conditions; however, no visually detectable melanin production was observed in the Δ*gdh*::*tyr1* strain when cultures were grown on glucose or mannitol for one week (Figure 5). Although the reduced-acidification phenotype eliminated the need for manual pH neutralization, these conditions did not produce detectable melanin, suggesting that factors other than medium acidification limit melanin production.

**Figure 6:**
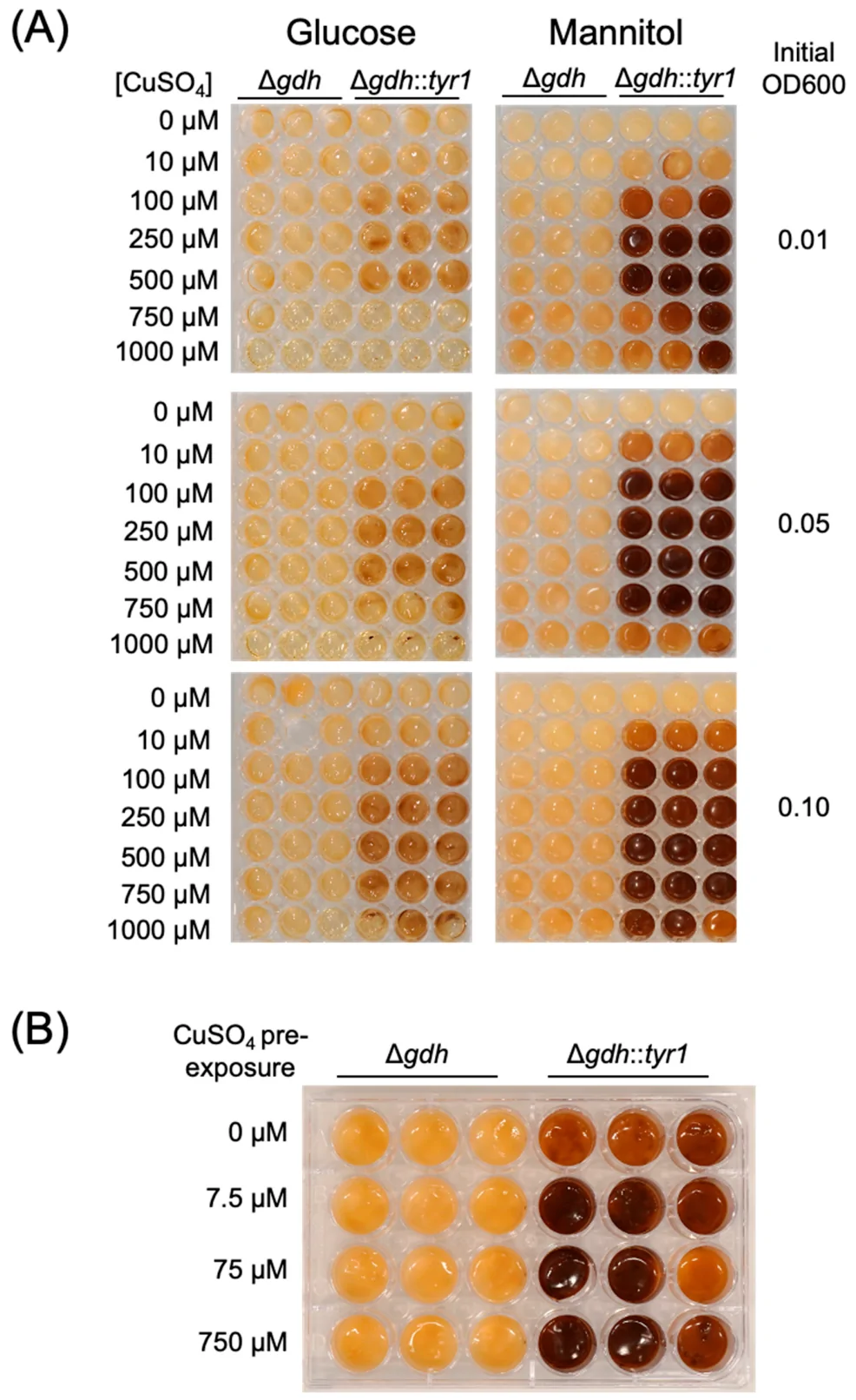
Optimization of one-step melanated bacterial cellulose production. (**A**) Melanation conditions were optimized by varying CuSO_4_ concentration, carbon source, and inoculation density. Triplicate cultures of the Δ*gdh* and Δ*gdh*::*tyr1* strains were grown in HS medium supplemented with L-tyrosine and CuSO_4_ as indicated. (**B**) To evaluate whether copper pre-exposure improved melanated pellicle production, triplicate Δ*gdh* and Δ*gdh*::*tyr1* starter cultures were pre-exposed to increasing concentrations of CuSO_4_ before inoculation into the optimized melanation medium.

Because previously reported conditions failed to produce detectable melanation and tyrosinase is a copper-dependent enzyme, we next optimized culture conditions by varying CuSO_4_ concentration, carbon source, and inoculation density (Figure 6A). Increasing CuSO_4_ concentrations progressively enhanced pellicle pigmentation in the Δ*gdh*::*tyr1* strain until concentrations that impaired pellicle formation were reached. Cultures grown on mannitol produced more robust pellicles and darker pigmentation than those grown on glucose. Inoculation density had comparatively little effect on melanation, although cultures inoculated at an OD_600_ of 0.1 consistently produced robust melanated pellicles over the widest range of CuSO_4_ concentrations. At concentrations higher than 750 µM, melanation was not consistent. Based on these results, HS medium containing mannitol, 0.5 g/L L-tyrosine, 750 µM CuSO_4_, and an initial OD_600_ of 0.1 was selected for all subsequent experiments.

Because the CuSO_4_ concentrations that elicited the greatest melanin production also inhibited pellicle formation, we next investigated whether pre-exposure to copper could improve copper tolerance while maintaining melanin production during pellicle formation (Figure 6B). Δ*gdh* and Δ*gdh*::*tyr1* cultures were pre-exposed to increasing concentrations of CuSO_4_ before inoculation into the optimized melanation medium. Pre-exposure to increasing CuSO_4_ concentrations enhanced pellicle pigmentation, with the greatest pigmentation observed following pre-exposure to 750 µM CuSO_4_. Accordingly, pre-exposure to 750 µM CuSO_4_ was incorporated into the final melanation protocol used for all subsequent experiments.

### 3.5. Characterization of Melanated Bacterial Cellulose

**Figure 7:**
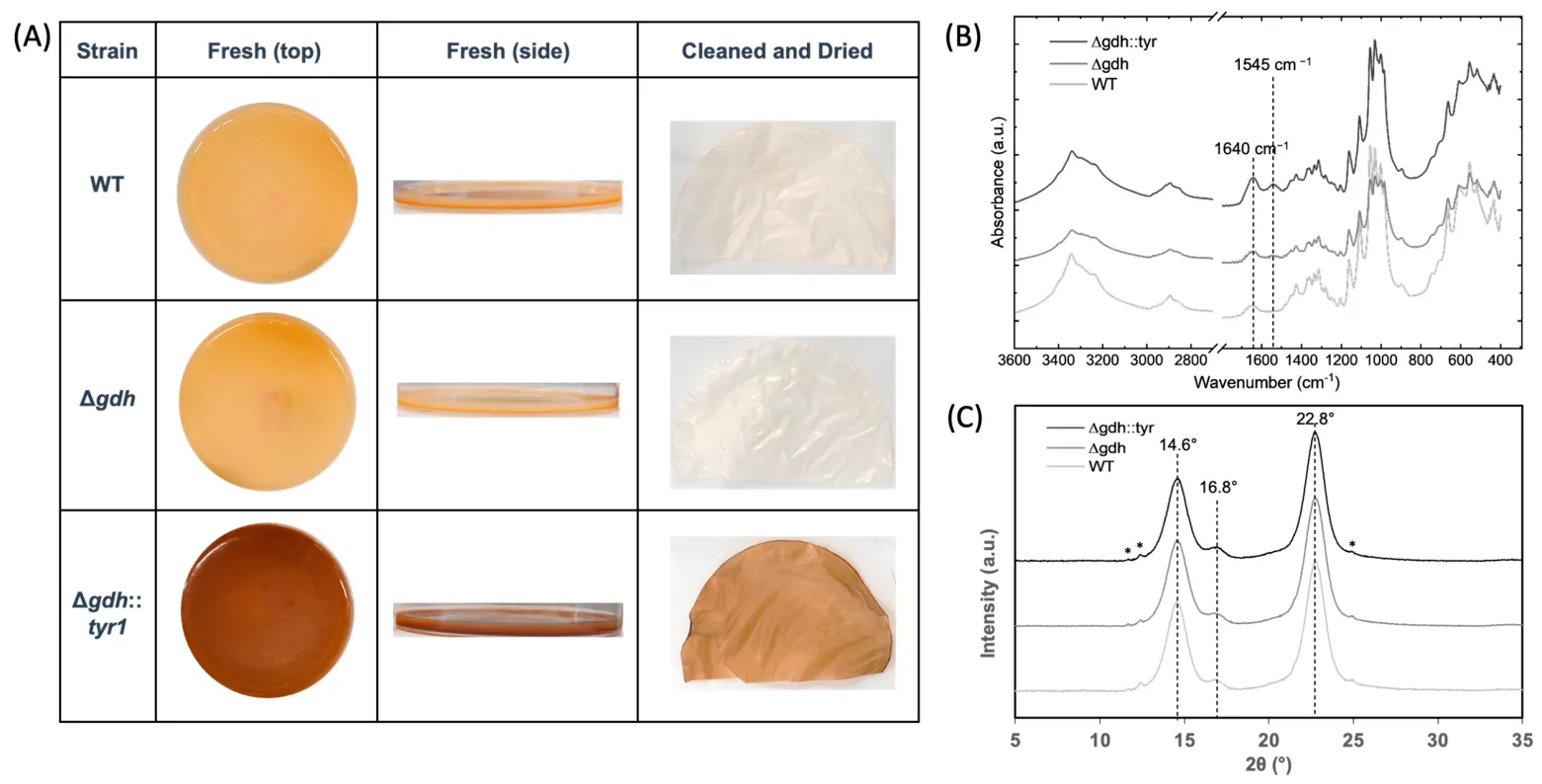
Characterization of melanated bacterial cellulose produced under optimized one-step melanation conditions. Pellicles produced by WT (n = 3), Δ*gdh* (n = 3), and Δ*gdh*::*tyr1* (n = 2) strains in 100 × 15 mm Petri dishes under optimized one-step melanation conditions were characterized. (**A**) Representative pellicles before NaOH treatment (top and side views) and after treatment and oven drying. (**B**) Molecular structure was observed using FTIR with representative spectra shown. The Δ*gdh*::*tyr1* sample shows evidence of melanin signal as denoted by the dashed lines at 1640 and 1545 cm^−1^. (**C**) Representative XRD patterns of NaOH treated and dried pellicles. Asterisks indicate peaks arising from the glass coverslip used during sample mounting.

To determine whether melanin production affected bacterial cellulose yield or structure, pellicles produced under the optimized one-step melanation conditions were characterized. Pellicles from the WT, Δ*gdh*, and Δ*gdh*::*tyr1* strains were produced at a 20 mL scale to generate sufficient material for downstream analyses. The Δ*gdh*::*tyr1* strain produced uniformly pigmented pellicles, and pigmentation was retained following NaOH treatment and oven drying (Figure 7A), consistent with previous reports demonstrating the stability of melanin incorporated into bacterial cellulose [14]. Pellicle dry weights were 82.4 ± 25.1 mg, 65.9 ± 16.1 mg, and 95.4 ± 8.6 mg for the WT, Δ*gdh*, and Δ*gdh*::*tyr1* strains, respectively, and did not differ significantly among strains, indicating that melanin production did not measurably affect bacterial cellulose yield (Table S4).

FTIR analysis of WT, Δ*gdh*, and Δ*gdh*::*tyr1* pellicles in Figure 7B shows no notable differences between the WT and Δ*gdh* strains, as expected. Polysaccharides, including cellulose, have characteristic peaks at ~3300 cm^−1^ (O-H stretching), ~2900 cm^−1^ (C-H stretching) [18], and ~1060 cm^−1^ (C-O stretching) [19]. The Δ*gdh*::*tyr1* strain exhibits peak differences attributable to the presence of melanin, as noted by the dashed lines at 1640 cm^−1^ and 1545 cm^−1^. The peak at 1640 cm^−1^ is indicative of C=N stretching, characteristic of melanin [20]. While WT and Δ*gdh* have a small peak at this position, this is due to C=C stretching in the cellulose structure. The peak at 1545 cm^−1^ is attributed to N-H bending and C-N stretching in melanin [21,22]. This peak is slightly shifted, likely due to changes in the chemical environment resulting from the incorporation of melanin into cellulose and the relatively low concentration of melanin compared to cellulose. Similarly, other characteristic peaks of melanin, such as N-H stretching around ~3400 cm^−1^ [22], cannot be elucidated due to the cellulose-to-melanin signal ratio.

XRD patterns of dried pellicles from all three strains exhibited the characteristic cellulose I reflections at 2θ values of 14.6, 16.8, and 22.8°, corresponding to the 1$\bar{1}$0, 110, and 200 crystal planes, respectively (Figure 7C). Crystallinity indices calculated using the Segal peak height method were 94.3 ± 1.9%, 90.5 ± 4.3%, and 95.3 ± 0.5% for the WT, Δ*gdh*, and Δ*gdh*::*tyr1* strains, respectively, and did not differ significantly among strains, indicating that melanin production did not measurably alter cellulose crystallinity.

## 4. Discussion

Genetic engineering of *Komagataeibacter* has enabled improved bacterial cellulose production and the development of novel functionalized materials; however, efficient strain engineering remains limited by practical challenges in screening candidate engineered transformants and in implementing acid-sensitive modifications. In this study, we demonstrate that the reduced-acidification phenotype resulting from *gdh* deletion provides a practical solution to both challenges. Specifically, reduced acidification enabled the development of a rapid, inexpensive, phenotype-based screening strategy for identifying engineered transformants and eliminated the need for manual pH neutralization, allowing one-step production of melanated bacterial cellulose. Together, these findings establish reduced acidification as a useful engineering phenotype that simplifies strain engineering and enables acid-sensitive functionalization of bacterial cellulose.

One practical challenge in engineering *Komagataeibacter* is efficiently identifying correctly engineered transformants. Although molecular confirmation remains essential for verifying genomic modifications, screening large numbers of transformants by colony PCR can become labor-intensive and costly, particularly when amplification is inconsistent. By exploiting the reduced-acidification phenotype associated with *gdh* deletion, we developed a simple phenotypic screening strategy that identifies transformants carrying the desired genomic modification. This approach enables direct whole-genome sequencing of candidate transformants, bypassing the need for intermediate PCR-based screening. This workflow is particularly useful for cellulose-producing strains because extracellular cellulose can interfere with rapid DNA-based screening methods and make colony PCR unreliable.

The effect of *gdh* deletion on bacterial cellulose production has been investigated previously, primarily in the context of improving cellulose yield from glucose. Prior studies have shown that disruption of membrane-bound glucose dehydrogenase reduces oxidation of glucose to gluconic acid, thereby improving conversion of glucose for bacterial cellulose production [12]. More broadly, bacterial cellulose yield and carbon conversion are strongly influenced by pH, buffer conditions, and carbon metabolism [23]. In the present study, deletion of *gdh* did not compromise cellulose production on mannitol, supporting its use as an engineering chassis under the conditions used for melanated bacterial cellulose production. This distinction is important because the objective of the present work was not solely to increase cellulose yield, but to use reduced acidification as an enabling phenotype for strain engineering and acid sensitive functionalization.

In addition to simplifying strain engineering, the reduced-acidification phenotype of Δ*gdh* expands the range of acid-sensitive modifications that can be implemented in *Komagataeibacter.* Previous studies demonstrated that production of melanated bacterial cellulose requires manual pH neutralization following pellicle formation because tyrosinase activity is inhibited under acidic conditions [14]. By coupling *gdh* deletion with the expression of *tyr1*, we eliminated the need for this post-production neutralization step, thereby enabling one-step production of melanated bacterial cellulose. Although optimization of culture conditions was required to achieve consistent melanation, manual pH neutralization was no longer necessary. This distinction is important because it shifts melanation from a post-growth processing step to a genetically enabled production phenotype. More broadly, this strategy could facilitate implementation of other acid-sensitive enzymes for bacterial cellulose modification, expanding the range of biologically mediated bacterial cellulose functionalization strategies.

Structural characterization demonstrated that melanin incorporation did not measurably alter bacterial cellulose yield or crystallinity, indicating that the one-step melanation strategy is compatible with native cellulose production. Under the optimized conditions used in this study, melanated bacterial cellulose production was achieved without detectable reduction in cellulose yield (dry weight) relative to the corresponding non-melanated control. Furthermore, retention of pigmentation following NaOH treatment is consistent with previous reports demonstrating stable incorporation of melanin within the cellulose matrix. Together, these findings suggest that reduced-acidification-enabled melanation can produce functionalized bacterial cellulose without substantially altering its underlying structural properties.

This work should be interpreted as a proof-of-concept strain engineering study rather than a fully optimized bioprocess. Accordingly, dry cellulose yield was measured as the primary production metric, but substrate conversion yield and volumetric productivity were not determined. Future process-focused studies should evaluate carbon conversion, production rate, media cost, reactor configuration, and scale-up performance under optimized cultivation conditions. Such studies will be necessary to determine the economic feasibility of reduced-acidification-enabled production of functionalized bacterial cellulose at the industrial scale.

## 5. Conclusions

This work demonstrates that the reduced-acidification phenotype resulting from *gdh* deletion provides a practical engineering strategy for *Komagataeibacter*, simplifying strain engineering while enabling one-step production of melanated bacterial cellulose. By combining phenotype-based screening with direct whole-genome sequencing, this workflow streamlines genome engineering without sacrificing definitive molecular confirmation. More broadly, reduced acidification expands the range of acid-sensitive bacterial cellulose functionalization strategies that can be implemented in *Komagataeibacter*.

## Figures and Tables

**Table 1:** Effect of *gdh* deletion on bacterial cellulose production by *K. sucrofermentans* grown with glucose or mannitol as the sole carbon source.

Carbon Source	Strain	Dry Weight (mg)
Glucose	WT	7.1 ± 3.5
	Δ*gdh*	11.1 ± 5.5
	*gdh* ^+^	4.1 ± 3.9
Mannitol	WT	85.5 ± 14.6
	Δ*gdh*	79.5 ± 1.8
	*gdh* ^+^	66.0 ± 20.3

Values represent the mean ± SD of three biological replicates. Statistical significance was determined by two-way ANOVA followed by Tukey’s multiple-comparison test (*p* < 0.05). Two-way ANOVA identified a significant effect of carbon source (*p* < 0.001).

**Table 2:** Validation of phenotype-based transformant identification by colony PCR and amplicon sequencing.

Method	Correct Genotype Assignment	Agreement with Phenotype
Hot Start Taq	6/20	6/6
Plant Phire Direct	15/20	15/15
Amplicon sequencing	15/15	15/15

## Data Availability

The original contributions presented in this study are included in the article/Supplementary Material. Further inquiries can be directed to the corresponding author.

## Supplementary Materials

The following supporting information can be downloaded at: https://www.mdpi.com/article/10.3390/polym18172099/s1, Figure S1. *K. sucrofermentans* WT growth and cellulose production on glucose and mannitol; Figure S2. Molecular validation of phenotype-based assignments shown in Figure 4; Figure S3. Phenotypic screening strategy applied to transformants of the Δ*gdh*::*tyr1* strain; Table S1. Oligo sequences and construct designs. Table S2. gBlock sequences; Table S3. Other oligos employed in this study; Table S4. Bacterial cellulose yields under optimized melanin-production conditions from 20 ml pellicle cultures.

## Abbreviations

The following abbreviations are used in this manuscript:

gdh	Glucose dehydrogenase
HS	Hestrim and Schramm
HS(G/M)T	HS with glucose/mannitol and tyrosine
Cel	Cellulase
RBS	Ribosomal binding site
Cm	Chloramphenicol
WT	Wild-type
pH	pH indicator dyes
FTIR	Fourier transform infrared spectroscopy
XRD	X-ray diffraction
gDNA	Genomic DNA
