# Supplementary material for: A Reduced-Acidification Phenotype Simplifies Strain Engineering in Komagataeibacter and Enables One-Step Production of Melanated Bacterial Cellulose
Source: Polymers (Basel). 2026 Aug 29;18(17):2099. doi: 10.3390/polym18172099 (PMC13568387; doi:10.3390/polym18172099)
Supplement: Supplementary file 1 [file polymers-18-02099-s001.zip › polymers-4503473-supplementary.pdf]

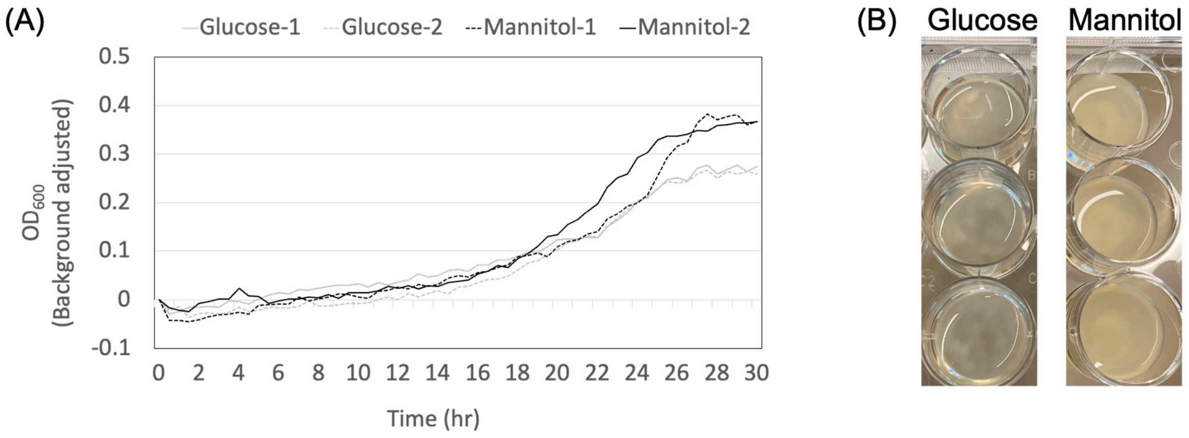

Figure S1. *K. sucrofermentans* WT growth and cellulose production on glucose and mannitol. (A) Duplicate 0.5 mL HSG-Cel and HSM-Cel volumes in a 48-well plate were spiked with WT at a starting OD_{600} of 0.0001 and measured for cell density every 30 minutes under aerobic conditions at 30 °C with 300 RPM shaking using a CLARIOstar plate reader (BMG Labtech, Ortenberg, Germany). Data shown represent signal above appropriate time = 0 hour values. (B) Triplicate 2mL HSG and HSM volumes in a 12-well plate were spiked with WT at a starting OD_{600} of 0.05 and incubated at 30°C for 5 days for pellicle formation. Resulting pellicles were visually observed for relative cellulose densities.

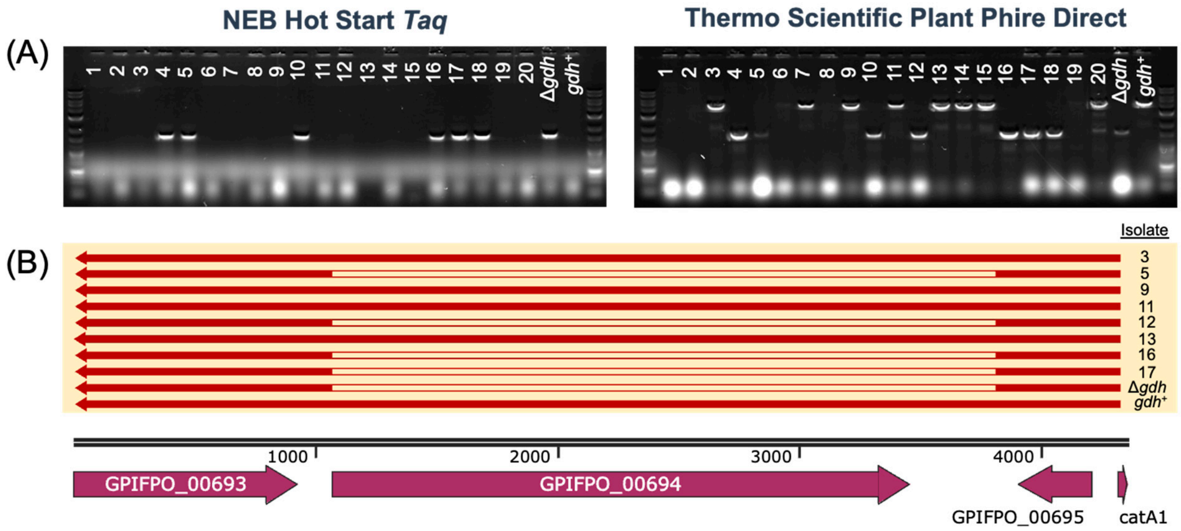

Figure S2. Molecular validation of phenotype-based assignments shown in Figure 4. (A) Biomass from isolated colonies was used as template for two commercial colony PCR kits according to manufacturer instructions using primers to anneal upstream of *gdh* (GPIFPO_00693) and within an introduced chloramphenicol resistance marker (*catA1*). (B) Strain identities were verified by sequencing of Plant Phire colony PCR amplicons.

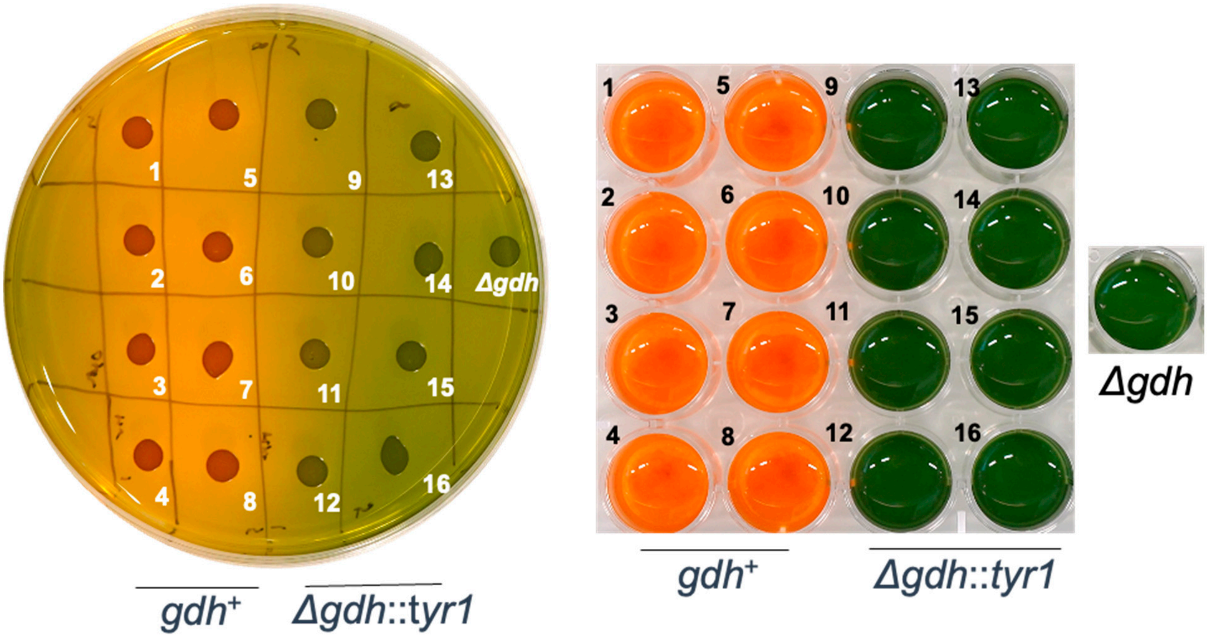

Figure S3. Phenotypic screening strategy applied to transformants of the Δ*gdh*::*tyr1* strain. Utility of pH-based method validated in **Figures 4** and **S1** was demonstrated in solid (left) and liquid (right) medium using fresh transformants of Δ*gdh*::*tyr1* in comparison to those of a *gdh*+ control strain.

Table S1. Oligo sequences and construct designs.

Oligos	Oligo Sequence	Template	PP Size (bp)
**pPETS_002 – Replace GmR in pBL_RE_024 with *CmR***			
O_PETS_005	cggggcgtaacaattcgttcaagccgag	Cm gBlock	692
O_PETS_006	tagcttccttcgttgctgctccataacatg		
O_PETS_007	agcagcaacgaaggaagctaaaatggagaaaaaaatcactggatataccaccg	pBL_RE_024	5418
O_PETS_008	gaacgaattgttacgccccgccctgcca		
**pPETS_003 – Create *gdh* KO plasmid**			
O_PETS_009	taactggatcaagcttggcgtaatcatgg	pPETS_002	2644
O_PETS_010	ggatggtgaactcgaattcactggccgtc		
O_PETS_011	tgaattcgagttcaccatcctgtcgtgtg	*Ks gDNA**	1089
O_PETS_012	cagcctacaccataagttttcctgattaccgtg		
O_PETS_013	aaaacttatggtgtaggctggagctgcttc	pPETS_002	1449
O_PETS_014	aggaaacactggtccatatgaatatcctccttagttc		
O_PETS_015	catatggaccagtgtttccttccccggc	*Ks gDNA*	1064
O_PETS_016	cgccaagcttgatccagttatcgaccggg		
**pPETS_051 – Create *gdh* rescue plasmid**			
O_PETS_066	gtgtaggctggagctgcttc	pPETS_014	3464
O_PETS_064	gtgctcgacgttgtcactg		
O_PETS_063	cagtgacaacgtcgagcac	pPETS_014	3683
O_PETS_052	cggtcttggcaaccaataggccgtactg		
O_PETS_053	cctattggttgccaagaccggcaacatc	*Ks gDNA**	1284
O_PETS_065	gaagcagctccagcctacaccatatcaccccgtatcatccgc		
**pPETS_014 – Insert *tyr1* into the *gdh* locus**			
O_PETS_028	cataagttttcctgattaccgtg	*Ks gDNA**	1069
O_PETS_032	ttcaccatcctgtcgtgtg		
O_PETS_025	cacggtaatcaggaaaacttatg	*tyr1 gBlock*	1070

O_PETS_026	gaagcagctccagccta		
O_PETS_027	gtgtaggctggagctgcttcagtgtttccttccccggct	pPETS_003	2510
O_PETS_035	agtgagcgcaacgcaatgatccagttatcgaccg		
O_PETS_033	cacacgacaggatggtgaaaaccaataggccgtactgc	pBL_RE_026	4638
O_PETS_036	atcattgcgttgcgctcactg		

* *Ks gDNA* = *K. sucrofermentans* genomic DNA

Table S2. gBlock sequences

gBlock	Sequence
CmR	aaggaagctaaaatggagaaaaaaatcactggatataccaccgttgatatatcccaatggcatcgtaaagaacatttt<br>gaggcatttcagtcagttgctcaatgtacctataaccagaccgttcagctggatattacggcctttttaaagaccgta<br>aagaaaaataagcacaagttttatccggcctttattcacattcttgcccgcctgatgaatgctcatccggagttccgt<br>atggcaatgaaagacggtgagctggtgatatgggatagtgttcacccttgttacaccgttttccatgagcaaactgaa<br>acgttttcatcgctctggagtgaataccacgacgatttccggcagtttctacacatatattcgcaagatgtggcgtgt<br>tacggtgaaaacctggcctatttccctaaagggtttattgagaatatgtttttcgtctcagccaatccctgggtgagt<br>ttcaccagttttgatttaaacgtggccaatatggacaacttcttcgcccccgttttcaccatgggcaaatattatacg<br>caaggcgacaaggtgctgatgccgctggcgattcaggttcatcatgccgtctgtgatggcttccatgtcggcagaatg<br>cttaatgaattacaacagtactgcgatgagtggcagggcggggcgtaa
tyr1	ttgacagctagctcagtcctaggtattgtgctagcattaaagaggagaaAAGAATGGGCAATAAATACCGCGTGCGTA<br>AGAATGTTCTGCACCTGACAGATACCGAGAAGCGTGACTTCGTGCGCACTGTACTGATTTTGAAAGAGAAGGGCATTT<br>ACGATCGTTACATCGCATGGCACGGCGCCGCGGGTAAGTTTCACACCCCGCCCGGTAGTGACCGTAACGCGGCGCACA<br>TGTCGAGTGCGTTCTTGCCTTGGCACCGCGAATATCTGCTGCGCTTTGAGCGCGATCTGCAATCGATTAACCCTGAGG<br>TGACTCTGCCGTACTGGGAGTGGGAAACCGATGCTCAAATGCAAGACCCTAGCCAGTCGCAGATCTGGAGCGCCGACT<br>TCATGGGCGGCAATGGCAACCCAATTAAGGACTTCATTGTAGACACGGGCCCGTTCGCTGCCGGCCGTTGGACAACCA<br>TTGACGAGCAGGGTAACCCGTCAGGCGGCTTAAAGCGCAACTTCGGTGCGACTAAGGAAGCCCCCACCCTGCCGACGC<br>GCGACGACGTGCTGAACGCACTTAAGATTACCCAATACGACACCCCACCCTGGGACATGACGTCCCAGAATAGTTTCC<br>GCAACCAACTCGAGGGTTTCATCAATGGCCCGCAACTGCATAACCGTGTGCATCGCTGGGTCGGTGGCCAAATGGGTG<br>TCGTCCCTACCGCGCCCAACGACCCGGTGTTCTTCCTGCATCATGCGAACGTTGACCGCATCTGGGCCGTGTGGCAGA<br>TCATCCACCGCAACCAGAATTACCAACCAATGAAGAATGGCCCGTTCGGCCAGAATTTCCGTGACCCAATGTATCCAT<br>GGAACACCACGCCTGAGGATGTAATGAATCACCGTAAACTGGGCTATGTTTATGACATCGAGTTGCGTAAGTCGAAGC<br>GCAGCTCTTGAccaggcatcaaataaaacgaaaggctcagtcgaaagactgggcctttcgttttatctgttgtttgtc<br>ggtgaacgctctc

Table S3. Other oligos employed in this study

Oligos	Oligo Sequence	PP Size (bp)	Description
PETS_ETH_107	ccatctccgctaactccgac	940	*E. coli* DH5α - PCR efficiency comparison (Figure 2)
PETS_ETH_109	ggttcttcgccgcagttcaatac		
O_PETS_11	tgaattcgagttcaccatcctgtcgtgtg	1080	*K. sucrofermentans* - PCR efficiency comparison (Figure 2)
O_PETS_17	tgaggctattcataagttttcctgattaccgtg		
O_PETS_11	tgaattcgagttcaccatcctgtcgtgtg	3582	Generate pPETS_003 linear fragment for transformation
O_PETS_16	cgccaagcttgatccagttatcgaccggg		
O_PETS_53	cctattggttgccaagaccggcaacatc	3758	Generate pPETS_051 linear fragment for transformation
O_PETS_16	cgccaagcttgatccagttatcgaccggg		
O_PETS_32	ttcaccatcctgtcgtgtg	4590	Generate pPETS_014 linear fragment for transformation
O_PETS_16	cgccaagcttgatccagttatcgaccggg		
O_PETS_11	tgaattcgagttcaccatcctgtcgtgtg	1611(Δ*gdh*)	PCR validation of phenotype-based screening (Figure S1)
PETS_ETH_4	caacggtggtatatccagtg	4354(*gdh+*)	

Table S4. Bacterial cellulose yields under optimized melanin-production conditions from 20 mL pellicle cultures.

Strain	Dry weight (mg)
WT	82.4 ± 25.1
Δ*gdh*	65.9 ± 16.1
Δ*gdh*::*tyr1*	95.4 ± 8.6

Values represent the mean ± SD of two (Δ*gdh*::*tyr1*) or three (WT, Δ*gdh*) biological replicates. Statistical significance was determined by two-way ANOVA followed by Tukey's multiple-comparison test ($P < 0.05$). No significant difference was determined between different strains.
